# Insights into pelvic venous disorders

**DOI:** 10.3389/fcvm.2023.1102063

**Published:** 2023-01-19

**Authors:** Kiara Rezaei-Kalantari, Guillaume Fahrni, David C. Rotzinger, Salah D. Qanadli

**Affiliations:** ^1^Department of Radiology, Rajaie Cardiovascular, Medical and Research Center, Cardio-Oncology Research Center, Iran University of Medical Sciences, Tehran, Iran; ^2^Department of Diagnostic and Interventional Radiology, Cardiothoracic and Vascular Division, Lausanne University Hospital and University of Lausanne, Lausanne, Switzerland

**Keywords:** pelvic venous disorders, varices, classification, treatment, radiology, interventional

## Abstract

Pelvic venous disorders (PeVD), sometimes referred to as pelvic congestion syndrome (PCS), widely impact affected patients–mainly young women’s quality of life, causing puzzling, uncomfortable symptoms sometimes requiring months or years to get an explanation, while simply remaining undiagnosed in other cases. Because pelvic pain is a non-specific symptom, an appropriate diagnosis requires a careful patient workup, including a correlation between history and non-invasive imaging. Invasive imaging is frequently required to confirm the diagnosis and plan treatment. Current therapeutic approaches principally rely on minimally invasive techniques delivered through endovascular access. However, while comprehensive descriptive classifications such as the symptoms-varices-pathophysiology (SVP) classification exist, universally accepted guidelines regarding therapy to apply for each SVP category are still lacking. This review strongly focuses on PeVD imaging and discusses available therapeutic approaches with regard to pathophysiological mechanisms. It proposes a new classification scheme assisting clinical decision-making about endovascular management to help standardize the link between imaging findings and treatment.

## 1. Introduction

It is estimated that up to 15% of women between the ages of 20 and 50 years are involved with varying degrees of pelvic venous disorder, though not all are clinically symptomatic (1–3). Furthermore, 30–45% of women with chronic pelvic pain (CPP) may have venous-related symptoms (4). Pelvic pain of venous origin is expressed chiefly as a vague pain with occasional escalation predominantly occurring after prolonged standing and walking and/or dyspareunia with prolonged post-coital aching. It is commonly a constant pain not connected to the menstrual cycle.

Lack of validated definitions and established imaging criteria, ambiguous cause and effect relationships, and presumed hypotheses based solely on small series have raised challenges for acceptance of the terms like pelvic congestion syndrome (PCS) by some medical societies. Pelvic venous disorder (PeVD) is the term that encompasses a range of venous disorders which result in CPP, mainly in women. CPP comprises many misleading historical so-called syndromes, including PCS, pelvic dumping syndrome, May-Thurner syndrome, and nutcracker syndrome, which may share similar symptoms with different but somewhat interrelated pathophysiologic mechanisms. Peculiar attention to PeVD would aim to raise both popular and healthcare awareness about the great number of women living with it, missing accurate diagnosis and thus lack proper care and curative treatment; A neglected morbidity based on WHO reviews may account for up to $2.8 billion in healthcare costs (5).

## 2. PeVD pathophysiology

The etiology of PeVD is not fully understood. It is multifactorial; genetic predisposition, anatomical abnormalities, and hormonal factors notably contribute to the pathogenesis. Half of the pelvic varice cases are attributed to genetic factors and have a positive familial history (6). Furthermore, congenital absence or dysfunction of valves are described (7), as well as other aspects, including chronic dilatation of veins causing venous wall inflammation and leading to aggravated reflux (8). Hormonal factors such as estrogen hyperstimulation are also hypothesized to take part in the pathophysiology (9), as is pregnancy due to associated increased vascular volume mainly distributed in the inferior vena cava system (10). Finally, anatomical variants can be the sole cause of pelvic varices, essentially due to proximal venous compression in the nutckracker phenomenon and iliac vein compression in the May-Thurner syndrome (11). The pelvic venous flow is primarily evacuated by women’s internal iliac veins as well as left and right ovarian veins, which are subsequently drained into the common iliac veins, inferior vena cava, and left renal vein, respectively. Venous hypertension and dilation due to vessel insufficiency (e.g., ovarian vein reflux, iliac vein reflux) or downstream stenosis/obstruction (e.g., iliac vein compression, renal vein compression) are hypothesized to be responsible for pain and fullness sensation by nociceptor activation and symptoms such as lower extremity and genital varicosities, pain and swelling, as well as flank pain and hematuria. In rare cases, epidural venous varices can develop, leading to neurological symptoms due to compressive radiculopathy (12, 13).

## 3. PeVD classification

### 3.1. SVP classification

Subdividing PeVD based on a valid and reliable discriminative classification instrument regarding the previously mentioned pathophysiologic entities and their clinical presentation would potentially lead to a more unified individual management and a more precise outcome measure. In 2021, the American Vein and Lymphatic Society international working group on PeVD released a multispecialty, intersocietal development of symptoms-varices-pathophysiology (SVP) classification (14). This classification comprises three components according to the presence and region of symptom (S), image-confirmed presence and region of dilated varices (V), and the pathophysiology (P) of the disorder. Thus, each individual patient’s classification is assigned as SVP_A,H,E_ (Table 1). In case of pelvic origin of lower extremity signs or symptoms, the SVP classification is used in combination with the Clinical-Etiologic-Anatomic-Physiologic (CEAP) classification (14, 15). The CEAP, revised in 2020, is a clinically useful and accepted classification for venous disorders, however, limited to the lower limbs only. In clinical practice, patients with S3b, S3c, or V3b disease in the SVP classification must also be classified with the CEAP scheme, which is a limitation of the SVP classification. Other limitations include the absence of patient representatives in the multidisciplinary panel of experts, the absence of widely accepted diagnostic criteria (16), difficulty representing variable and complex hemodynamic and clinical features, and the absence of proposed management.

**TABLE 1:** Symptoms-varices-pathophysiology (SVP) classification.

**Symptoms (S)**	
S_0_	No symptoms
S_1_	Renal symptoms of venous origin
S_2_	Chronic pelvic pain of venous origin
S_3_	Extra-pelvic symptoms of venous origin
a	Localized symptoms associated with veins of the external genitalia
b	Localized symptoms associated with pelvic origin non-saphenous veins of the leg
c	Venous claudication
**Varices (V)**	
V0	No abdominal, pelvic, or pelvic origin extra-pelvic varices
V1	Renal hilar varices
V2	Pelvic varices
V3	Pelvic origin extra-pelvic varices
a	Genital varices (vulvar varices and varicocele)
b	Pelvic origin lower extremity varicose veins arising from the pelvic escape points and extending into the thigh
**Pathophysiology (P)**	
Anatomy	Inferior vena cava; left renal vein, gonadal vein, common iliac vein; external iliac vein; internal iliac vein; pelvic escape vein
Hemodynamics	Obstruction (O); reflux (R)
Etiology	Thrombotic (T); non-thrombotic (NT), congenital (C)

### 3.2. Proposed management-oriented classification

**FIGURE 1:**
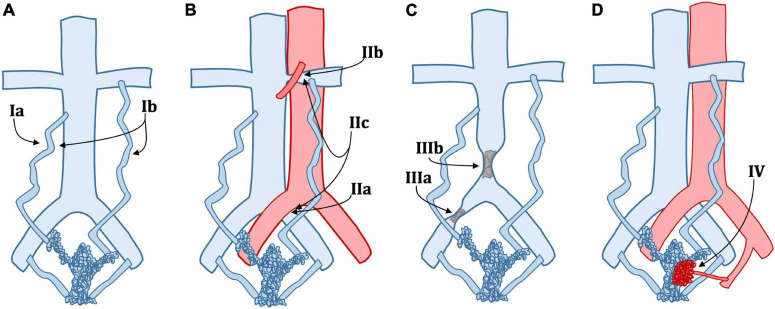
Schematic classification of pelvic venous disorders (PeVD). **(A)** Type Ia: unilateral venous insufficiency, Type Ib: bilateral venous insufficiency; **(B)** Type IIa: May-Thurner syndrome, Type IIb: nutcracker phenomenon, Type IIc: both IIa and IIb; **(C)** Type IIIa: common iliac vein obstruction, Type IIIb: inferior vena cava obstruction; **(D)** Type IV: arteriovenous malformation or fistula. Type IId (extrinsic compression), IIIc (portal hypertension), and V (nutcracker Syndrome) are not displayed.

The SVP classification helps define the symptomatic, anatomic, and etiologic aspects of PeVD. It gives a straightforward way to communicate between the radiologist and the clinician to provide a full picture of patients’ condition comprehensively. However, SVP does not give precise insights into treatment. There is a need for a more clinically and management-oriented classification to help the diagnostic radiologist better communicate with the interventional radiologist. Table 2 details a new classification based on patient management rather than clinical profile or anatomical factors. A graphical representation of the same classification is provided in Figure 1.

**TABLE 2:** Proposed management-oriented classification of pelvic venous disorders (PeVD), divided into five types. Each type or subtype is associated with a specific treatment.

Type	Description	Treatment^1^
**I**	**Venous insufficiency**	
Ia	Unilateral venous insufficiency	Embolization
Ib	Bilateral venous insufficiency	Embolization
**II**	**Venous compression**	
IIa	May-Thurner syndrome	Stenting ± embolization
IIb	Nutcracker phenomenon	Embolization ± stenting
IIc	May-Thurner syndrome and nutcracker phenomenon	Stenting, embolization
IId	Other extrinsic venous compression	Stenting
**III**	**Venous obstruction**	
IIIa	Common iliac vein obstruction	Stenting
IIIb	Inferior vena cava obstruction	Stenting
IIIc	Portal hypertension	Stenting
**IV**	**Arteriovenous malformation or fistula**	Embolization
**V**	**Nutcracker syndrome**	Stenting or surgery

^1^Treatment warranted based on symptoms severity and patient condition. Balance risk/benefit ratio should be carefully assessed.

Our proposed classification is divided into five main types. Type 1 describes PeVD due to venous insufficiency, which can be unilateral (Ia) or bilateral (Ib) and is treated with unilateral or bilateral embolization. Type 2 comprises all venous compression disorders, such as May-Thurner syndrome (IIa), nutcracker phenomenon (IIb), combined May-Thurner and nutcracker phenomenon in the same patient (IIc), and other extrinsic venous compression (IId), all subtypes requiring either embolization, stenting or a combination of both. Type 3 is due to all types of venous obstruction and is subdivided into common iliac vein (IIIa), inferior vena cava (IIIb), and portal hypertension (IIIc), all treated with stenting. Type IV includes all PeVD due to arterio-venous malformations or fistulae, which are treated with embolization. Type V is reserved for nutcracker syndrome. It is essential to differentiate the nutcracker phenomenon (compression of the left renal vein and distal venous distention) from nutcracker syndrome, where the left renal vein hypertension results in clinical symptoms such as hematuria or flank pain (17), which can be either treated with stenting or surgery. It is important to note that while our proposed classification links each entity to the most commonly appropriate treatment, it should be taken as a general framework over which clinical and individual aspects must be taken into consideration. For example, careful tumoral characteristics and invasion assessment should be made in IId lesions to ensure that stenting is an appropriate approach. Also, the amount of pain, as well as the severity of other symptoms should be a major point in deciding whether to perform an interventional approach. Finally, anatomical variations, such as duplicated IVC, extra iliac vessels, variant drainage patterns, and shortening or absence of veins, are not included in this classification due to their rarity but could critically change the treatment and must absolutely be taken into account.

## 4. Role of imaging in PeVD

A variety of imaging modalities, including ultrasound, venography [with or without intravascular ultrasound (IVUS)], computed tomography (CT), and magnetic resonance imaging (MRI), have been used in the evaluation of pelvic venous disorder. It is critical to remember that due to the still lacking definitive anatomic and hemodynamic criteria for the PeVD spectrum, any non-invasive imaging assessment alone could not be considered a criterion for intervention and should be interpreted with caution after prudent correlation with the clinical symptoms.

### 4.1. Greyscale and Doppler ultrasound

Several studies have stated various imaging criteria for pelvic varicosis and congestion, among which pelvic ultrasound (US), either transabdominal or transvaginal, is the widely practiced exam for initial evaluation (3, 18). US has the power not only to evaluate the venous diameter but also real-time assessment of venous insufficiency and reflux during maneuvers. Ovarian vein diameter is mainly used as a marker for retrograde flow. Pelvic varices have been found to be present in all patients with a left ovarian vein diameter > 6 mm (19), and evidence of a dilated, tortuous vein showing ≥ 5 mm diameter around the ovary and uterus has been the most cited US finding (1). Meanwhile, studies (20–22) revealed that the mere reliance on the diameter is insensitive for reflux detection and others (1) declared positive predictive values of 71.2, 83.3, 81.8, and 75.8% for ovarian vein diameters of 5, 6, 7, and 8 mm, respectively. In practice, four major indicators are used to diagnosis PeVD, summarized in Table 3. The first is venous diameter, with the above-discussed 5 mm threshold for periovarian and periuterine veins. The second is the number of dilated veins, with a threshold of more than four dilated (>4 mm) periovarian and periuterine veins. The third is the presence of venous connections (*trans*-uterine veins) between left and right uterine veins. The fourth is alteration of venous flow during Valsalva maneuver.

**TABLE 3:** Few of the mentioned ultrasound (US) criteria for patients with pelvic varicosis.

Ultrasound findings in PVeD
Dilated, tortuous veins (**≥**5 mm) around the ovary and uterus
More than four dilated tortuous veins (>4 mm) around the ovary and uterus
Dilated *trans*-uterine veins (arcuate and/or myometrial veins) connecting the left and right uterine veins
Disappearance, altered, or reversed flow with Valsalva maneuver

As mentioned above, the imaging criteria are neither sensitive nor specific for confirmation. Dos Santos et al. showed no significant difference between the diameters of competent and refluxing ovarian veins in transvaginal ultrasound (TVS), as vessel diameter alone was only 56% accurate in reflux identification (21). Park et al. found *trans*-uterine crossing veins and flow reversal during Valsalva maneuver in only about one-fourth of symptomatic compared to about 9% of an asymptomatic control group (1). Additionally, in a recent study (23) using TVS, there were myometrial crossing veins in 33.3%, reverse or altered flow during Valsalva in 25%, and the most prominent pelvic vein ≥ 8 mm in 25% of the venography-negative control group. The issue becomes more complicated, knowing that patient position and maneuvering impact the imaging accuracy for detecting pelvic venous pathology. While CT and MR imaging are done in the supine position, investigators have used diverse technical approaches (e.g., supine, 30° to 45° reverse Trendelenburg position, semi-erect, upright) (1, 20, 21, 24), and there is still no agreement on which posture it should be performed. It is worth mentioning that the existence of anatomical variations in the pelvic venous network and the collapse of the varicose veins due to bladder filling during transabdominal ultrasound exams might increase the difficulty of its standardization. Identifying retrograde flow as the basis of venous reflux has been described in the left ovarian vein of symptomatic pelvic varices compared to 25% of controls (19). Variable duration and patterns of retrograde flow for detection and documentation of pelvic reflux have been tried and suggested (16, 19, 21, 25). It can be seen spontaneously or in response to a Valsalva maneuver during ultrasound, invasive venography, or time-resolved MRI.

### 4.2. Cross-sectional imaging (CT, MRI)

Cross-sectional imaging of the abdomen and pelvis is a commonly utilized examination in evaluating female abdominal and/or pelvic pain. Owing to its widespread use, CT is most likely to be the imaging modality that reveals dilated gonadal veins. It provides an evaluation of parametrial varices, and related findings, such as nutcracker anatomy or any retroperitoneal lesion that may be causing venous congestion. Like in ultrasound, an ovarian vein diameter > 5 mm is generally considered dilated on a CT scan (4, 26, 27). At the same time, studies suggested thresholds for a normal gonadal vein diameter, ranging from 2.6 to 7 mm according to parity status (28, 29). Diagnostic findings proposed by Coakley et al. for cross-sectional imaging (both CT and MRI) include ≥ 4 ipsilateral parauterine veins of varying size, one of them showing >4 mm in diameter or the ovarian vein measuring > 8 mm. However, these cut-off diameters also differ between studies (4, 6, 30, 31). Visualizing contrast medium flowing back from the left renal to the left ovarian vein on a CT scan is a common finding during the renal corticomedullary phase, seen in approximately 40% and often parous asymptomatic women (28, 32). A paper divided reflux among these patients into three grades according to the degree of retrograde filling; (a) limited to the ovarian vein, (b) extending to ipsilateral parauterine veins, and (c) crossing the pelvic midline to the contralateral plexus, respectively. Most of their patients with grade III reflux were multiparous (33). In patients with nutcracker syndrome, a statistically significant reduction of renal vein diameter compared to the control group is noted on CT angiography (34). Moreover, a venography pressure gradient of 3 or more mmHg across the stenotic point has been associated with the development of hematuria (35–37). However, ≥50% diameter narrowing of the left renal and iliac veins during their course between the large vessels and vertebral body are common findings on all imaging modalities, seen in more than half and up to one-third of asymptomatic cases, respectively. In addition, the gradient across a significant renal or iliac vein obstruction in patients with collaterals may not be greater than the 3 mmHg venography gradient promoted to suggest a significant lesion (38). A systematic review (18) on the non-invasive diagnostic tools for PCS concluded that CT provides more limited venous flow information than MRI and lacks sufficient scientific validation. In fact, its radiation hazards are a drawback and remain a concern for most young women of fertile age. It should be reminded that CT scan should be used with caution, especially since these young patients will have further exposure to ionizing radiation during endovascular therapy. On the contrary, MRI [specifically phase-contrast velocity mapping (PCVM) and time-resolved imaging (TRI) sequences] has a high reported sensitivity of 67% to more than 88%. At the same time, MRI provides accurate anatomical information, flow direction in the ovarian vein (39–41), and the potential for exclusion of differential diagnoses such as endometriosis, gastrointestinal or musculoskeletal pathology, or tumors with no radiation hazard in women of childbearing age (42–45). Yang et al. (45) compared TRI with conventional venography. They demonstrated that it is an outstanding non-invasive diagnostic tool for determining the level of ovarian venous reflux with no significant difference from conventional venography. However, the lack of standardized criteria and their limited availability has complicated its recommendation.

In an attempt to further systematize and boost the diagnostic and therapeutic approach, Szary et al. (46) described a hemodynamic-anatomical classification for the grading of ovarian veins insufficiency–as one of the most common reasons for pelvic venous insufficiency (PVI)–based on color-Doppler ultrasound, CT, and MR venography. Their 4-grade scale took into account various factors comprising the mean diameter and incompetence of the ovarian veins, the mean diameter of the internal iliac veins and their branches, maximum distension of the para-uterine venous plexuses on both sides, the presence of collateral venous circulation and occurrence of pelvic venous anastomoses (Table 4). Those subjected to groups III and IV were proposed to require bilateral embolization. Authors claimed that such classification provided ease to be used in everyday clinical practice and could facilitate communication between the specialists dealing with the pathology.

**TABLE 4:** Grading classification for ovarian veins system insufficiency proposed by Szary et al. (øLOV/øROV) left or right ovarian vein diameter (mm); (incLOV/incROV) left or right ovarian vein incompetence (+ or -); (lPUV/PUV) left or right para-uterine veins (mm); (bLIILV/bRILV) branches of left or right internal veins (mm).

	øLOV	incLOV	lPUV	bLIILV	øROV	incROV	rPUV	bRIILV
GI	<6	(-/ +)	<5	<5	<5	(-)	<5	<5
GI/II	6–6.5	(+)	<5.5	<5.5	<5	(-)	<5	<5
GII	<7	(+)	<6.5	<6	<5.5	(-)	<5.5	<5.5
GII/III	7.5–8	(+ +)	<7	<7	<6	(-/ +)	<6.5	<6
GIII	>8	(+ +)	7–8	<7.5	<7.5	(+ / + +)	<7	<6.5
GIV	>10	(+ + +)	>8	>8	>8	(+ + +)	<7.5	>7

### 4.3. Venography

Conventional venography is still the gold standard for establishing the diagnosis of PeVD. Since it is an invasive examination, venography should be kept for patients with prior non-invasive imaging when intervention is planned (18, 47–50). Usually, selective ovarian and iliac catheter venography is performed for confirmation. However, alternative venographic methods have been published, such as direct percutaneous needle injection of contrast into the uterine myometrium for fluoroscopic assessment of the venous plexus (51). The known Beard’s criteria consist of three measures, and each is scored from 1 to 3, with the sum ≥ 5 considered as confirmative for pelvic venous disorder. These three components include: the maximum detected diameter of the ovarian vein (5–8 mm moderate, >8 mm severe, while less than 5 mm is considered normal), the time for contrast washout (0, 20, and 40 s), and intensity of congestion (normal: straight and small veins, moderate: tortuous veins, severe: highly tortuous and dilated veins). The criteria have a reported sensitivity and specificity of about 90% (47). A reduction of 50% or more cross-sectional area on IVUS or diameter in venography in the context of attributable pelvic or lower extremity symptoms has been widely considered significant iliac vein stenosis, and a number of corresponding ultrasound criteria for detection have also been developed (24, 52). Nevertheless, there are remaining uncertainties (53–55), and the value may differ between the patients (56). The VIDIO trial, which evaluated a > 4-point reduction in the revised Venous Clinical Severity Score between baseline and 6 months as an indicator of clinically meaningful improvement, demonstrated that a cross-sectional area decrease of >54% by IVUS examination had the highest sensitivity (83% sensitivity, 47% specificity). In contrast, a greater than 52% reduction in diameter by venography had the highest specificity (50% sensitivity, 71% specificity). As expected, the post-stenting clinical improvement threshold was higher for non-thrombotic cases (53).

## 5. PeVD treatment

A unified and optimal treatment option for patients with PeVD is lacking partly due to previous non-randomized cohort studies with varying treatment techniques applied to diverse symptoms and etiologies.

### 5.1. Compression stockings and physiotherapy

The usage of elastic compression stockings as a conservative treatment for even a short period of 2 weeks resulted in diminished pain, dyspareunia, swelling, heaviness, and discomfort in more than 80% of patients (57, 58). Though wearing it for a long time is bothersome, discouraging and non-compliance is a major issue in this kind of treatment. Physiotherapy has been proposed, but efficacy evidence is lacking (59, 60).

### 5.2. Pharmacological and hormonal

Medications such as medroxyprogesterone acetate (MPA), etonogestrel implants, non-steroidal anti-inflammatory drugs (NSAIDs), and gonadotropin-releasing hormone (GnRH) agonists for PeVD have been shown to be effective for short-term symptom relief. However, they have been still discouraging due to the lack of data determining convincible long-term efficacy (61–63). However, micronized purified flavonoid fraction (MPFF), a venoactive drug, has shown promise in reducing pain and heaviness due to pelvic varicose veins and improved venous outflow within the first weeks of treatment (64–67).

### 5.3. Surgery

Data regarding surgery for PeVD is controversial. It is associated with a more extended hospital stay and, depending on the organ treated, has a greater mortality risk compared with endovascular therapy (68). In addition, about one/fifth to one/third of patients experience recurrence or residual pain after hysterectomy (69). Hysterectomy with either unilateral or bilateral salpingo-oophorectomy resulted in significantly lower pain relief compared with endovascular embolization of the ovarian vein (70). In the case of nutcracker syndrome with hematuria, gonadocaval bypass surgery has been proven an effective treatment option (71). Gonadal vein resection has also been used effectively to treat gonadal vein incompetence in a study involving 57 patients (72).

### 5.4. Endovascular therapy

#### 5.4.1. Embolization

Endovascular embolization, also called “vaso-occlusive therapy,” is recommended to treat PCS with a 2B level of evidence by the Society for Vascular Surgery and American Venous Forum (73). The techniques and the embolic agents vary in publications (16, 74). The standard approach uses coils, plugs, and sclerotherapy, either alone or in combination. Coils have been shown to improve clinical symptoms, with long-term effects and low complication rates, while having the disadvantage of reintervention needed in up to 10–30% of patients (75–78). Plugs are an excellent alternative, as effective and safe as plugs but can often only be effective if the largest plug size is used due to the relatively large size of veinous dilatations (79–81). Percutaneous sclerotherapy is used more as a treatment for vulvar and cutaneous varices secondary to pelvic venous congestion, with good results (82–85).

**FIGURE 2:**
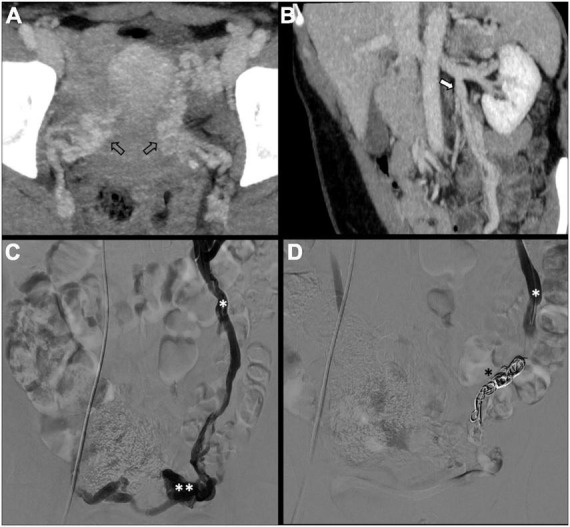
Type Ia pelvic varices in a 48 yo female due to unilateral venous insufficiency. **(A,B)** Computed tomography (CT) phlebography shows pelvic varices (open arrows) with a dilated left ovarian vein (white arrow). **(C,D)** Conventional phlebography and CT phlebography show pelvic varices (double white asterisks) with a dilated left ovarian vein (single white asterisk), treated with multiple coils (single black asterisk).

**FIGURE 3:**
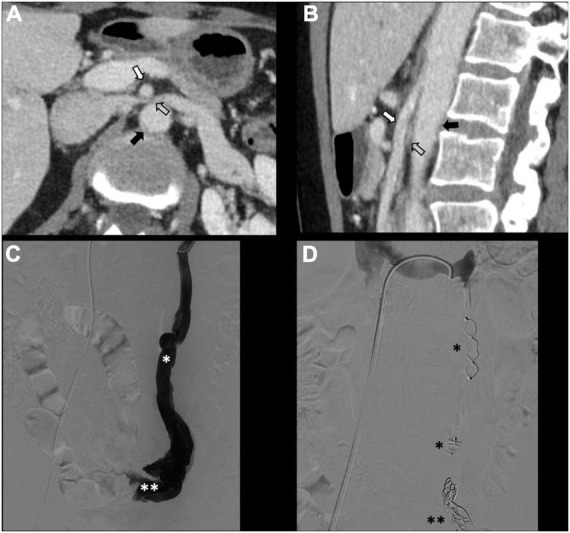
Type IIb pelvic varices in a 54 yo female due to nutcracker syndrome. **(A,B)** Computed tomography (CT) phlebography shows the left renal vein (open arrow) compressed by the mesenteric artery (white arrow) and aorta (black arrow). **(C,D)** Conventional phlebography shows dilated left ovarian vein (single white asterisk) with left-sided pelvic varices (double white asterisks), treated with multiple plugs (single black asterisk) and coils (double black asterisks).

**FIGURE 4:**
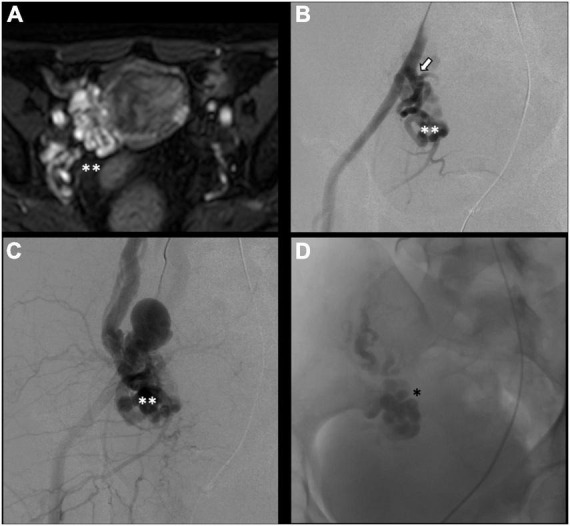
Type IV pelvic varices in a 44 yo female due to arteriovenous fistula. **(A)** Magnetic resonance imaging (MRI) shows right-sided pelvic varices (double white asterisks) with flow voids and early opacification in arterial phase. **(B–D)** Conventional phlebography shows early arterial opacification of varices (double white asterix) due to a fistula from the left internal iliac artery (white arrow), treated with Onyx embolization (single black asterisk).

There is no consensus on how to report results, and the final results are very heterogeneous. Though, few studies, systematic reviews, and randomized trials have displayed no remarkable differences between embolization materials in terms of outcome (16, 79, 86). Furthermore, studies have reported an overall rate of complete, excellent, or moderate improvement of 75% at 4–8 weeks and more than 80% at an average of 45 months post-procedure, regardless of the technique or agent used (16, 87–90). Figure 2 shows an example of type Ia pelvic varices and subsequent vaso-occlusive therapy by means of multiple endovenous coils. Endovascular vaso-occlusive therapy is also applicable in the nutcracker phenomenon, with reported improvement in 56 to 98% of patients (17). Figure 3 shows an example of nutcracker phenomenon corresponding to a IIb pelvic varix stage. In the case of arteriovenous fistula (type IV pelvic varices), distal embolic efficacy can be achieved with liquid agents, as shown in Figure 4.

Unilateral or bilateral embolization is still under debate. Some studies found that the clinical result difference was not statistically significant (49). Nevertheless, others recommend performing complete embolization (i.e., both ovarian and iliac veins) for all since they had a very high (near 95%) and sustained clinical improvement at a mean follow-up of almost 5 years (86, 91). It would be reasonable to consider embolization for the veins that are indeed insufficient. Maleux et al. did bilateral embolization only for cases with bilateral ovarian vein insufficiency and found no statisticaly significant difference in clinical outcomes between the two groups (92). This is the same as the approach that Szary et al. (46) used for their patient treatment. In terms of clinical response, younger patients, especially those in their 20 s, have smaller ovarian vein diameters, and patients with low-emotional expression usually have a shorter duration and better sense of improvement than their older and low-stress tolerance counterparts (93, 94).

#### 5.4.2. Stenting

**FIGURE 5:**
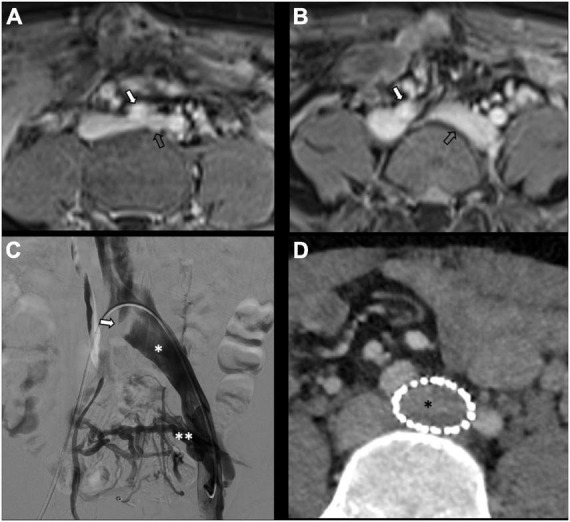
Type IIa pelvic varices in a 30 yo male due to May-Thurner syndrome. **(A,B)** MR phlebography shows left common internal iliac vein (open arrow) compressed by the right common iliac artery (white arrow). **(C,D)** Conventional and computed tomography (CT) phlebography show dilated left common iliac vein (single white asterisk) with left-sided pelvis varices (double white asterisks), treated with stenting (single black asterisk).

Although studies show benefits from ovarian vein embolization procedures, questions remain regarding the need for left common iliac vein stenting for non-thrombotic iliac vein lesions. It is obvious that removal of the obstruction should be undertaken whenever stenosis is present, representing a hemodynamically significant problem (95). In an article published by Lakhanpal et al., in PVI due to iliac vein stenosis, stenting alone led to the complete resolution of symptoms in 56% of patients (93). Santhoshi et al. moved a step forward in their non-randomized retrospective study and published significant and possibly greater improvements in VAS pain scores after non-thrombotic iliac vein compression stenting compared to embolization alone or staged stenting after embolization (95). Nevertheless, the majority (80%) of their patients possessed significant (>50%) iliac vein stenosis on IVUS, which led them to claim that the incidence of iliac vein outflow obstruction in PVI is greater than previously reported. On the contrary, Gavrilov et al. showed that the sole stenting of the left iliac vein -without ovarian vein embolization- resulted in symptom relief in only about 17% of PeVD patients due to May-Thurner Syndrome (96). Figure 5 shows an example of left common iliac vein stenting due to type IIa pelvic varices in a young male with May-Thurner syndrome. In the case of nutcracker phenomenon, while embolization is a preferred choice of treatment, stenting can also be considered, however, with a risk of stent migration (97). Regarding thrombotic and stenotic venous lesions, stenting has been proven effective with long-term patency rates, low morbidity, and migration rates, the main complication being intrastent thrombosis (98–100). As the thrombus ages, it tends to incorporate itself in the venous wall, forming a more chronic collagen-predominant structure that could be more difficult to successfully stent (101). Nonetheless, even in chronically thrombosed veins, stenting remains a low-risk procedure with acceptable long-term patency rates (102).

## 6. Conclusion

We discussed the pathophysiology, classification, imaging, and treatment of PeVD. We proposed a new management-oriented classification system to address the current lack of a comprehensive system regrouping the mechanisms responsible for the occurrence of PeVD–which is key to selecting proper treatment, determining homogenous patient populations, broadening clinical communications, fostering the development of clinical trials, and making literature results interpretation unchallenging.

## Author contributions

KR-K wrote the main sections of the manuscript. GF wrote multiple sections and designed tables and figures. DR wrote multiple sections, contributed to drafting the manuscript, and coordinated writing and revisions. SQ contributed to the concept design, new classification design, manuscript drafting, and review of all sections. All authors read and approved the manuscript.
